# Deoxycholate, an Endogenous Cytotoxin/Genotoxin, Induces the Autophagic Stress-Survival Pathway: Implications for Colon Carcinogenesis

**DOI:** 10.1155/2009/785907

**Published:** 2009-05-10

**Authors:** Claire M. Payne, Cheray Crowley-Skillicorn, Hana Holubec, Katerina Dvorak, Carol Bernstein, Mary Pat Moyer, Harinder Garewal, Harris Bernstein

**Affiliations:** ^1^Department of Cell Biology & Anatomy, College of Medicine, University of Arizona, Tucson, AZ 85724-5044, USA; ^2^Arizona Cancer Center, University of Arizona, Tucson, AZ 85724-5044, USA; ^3^INCELL Corporation, San Antonio, TX 78249, USA; ^4^Department of Internal Medicine, College of Medicine, University of Arizona, Tucson, AZ 85724-5044, USA; ^5^Southern Arizona Veterans Affairs Health Care System, Tucson, AZ 85723, USA

## Abstract

We report that deoxycholate (DOC), a hydrophobic bile acid associated with a high-fat diet, activates the autophagic pathway in non-cancer colon epithelial cells (NCM-460), and that this activation contributes to cell survival. The DOC-induced increase in autophagy was documented by an increase in autophagic vacuoles (detected using transmission electron microscopy, increased levels of LC3-I and LC3-II (western blotting), an increase in acidic vesicles (fluorescence spectroscopy of monodansycadaverine and lysotracker red probes), and increased expression of the autophagic protein, beclin-1 (immunohistochemistry/western blotting). The DOC-induced increase in beclin-1 expression was ROS-dependent. Rapamycin (activator of autophagy) pre-treatment of NCM-460 cells significantly (*P* < .05) decreased, and 3-MA (inhibitor of autophagy) significantly (*P* < .05) increased the cell loss caused by DOC treatment, alone. Rapamycin pre-treatment of the apoptosis-resistant colon cancer cell line, HCT-116RC (developed in our laboratory), resulted in a significant decrease in DOC-induced cell death. Bafilomycin A_1_ and hydroxychloroquine (inhibitors of the autophagic process) increased the DOC-induced percentage of apoptotic cells in HCT-116RC cells. It was concluded that the activation of autophagy by DOC has important implications for colon carcinogenesis and for the treatment of colon cancer in conjunction with commonly used chemotherapeutic agents.

## 1. Introduction

Colon cancer is the second leading cause of cancer deaths among men and women combined in the United States, Australia, and Europe and is a major problem in many other countries. Approximately 50% of colorectal cancers are attributed to dietary factors [1]. A typical Western diet, high in fat and low in fiber, has been shown to contribute to the development of colon cancer in epidemiologic and animal studies [2]. Bile acids/salts, present in high concentration in the feces of patients on a high fat/low fiber diet [3], have been associated with colon cancer risk [4]. The most common bile acid present in the human feces is deoxycholic acid (DOC) [5], a hydrophobic bile acid. DOC is a promoter of colon cancer [2], and also a genotoxic carcinogen [6–8], and may be responsible for initiating gastrointestinal cancers (reviewed by Bernstein et al. [9]). However, the mechanism by which hydrophobic bile acids act in progression to colon cancer is unclear.

Hydrophobic bile acids are known inducers of at least five stress-response pathways in gastrointestinal cells, including ER stress [10], oxidative stress [6, 11–13], nitrosative stress [14, 15], mitochondrial stress [10–13, 16], and DNA damage [6, 17–19]. Some of these bile acid-induced cellular stresses may ultimately lead to cell death by mechanisms that include both apoptosis [20, 21] and necrosis [16]. Hydrophobic bile acids also promote colon cancer. In addition, they may act as carcinogens [6, 7] and/or select for outgrowth of clones of mutant cells resistant to bile acid-induced cell death. One of the cell survival pathways activated in response to bile acid exposure is the NF-*κ*B stress-response pathway [11, 20, 22, 23]. Persistent activation of NF-*κ*B causes cells to become apoptosis-resistant, and such cells tend to acquire mutations, some of which may contribute to colon cancer.

Another important cell survival pathway is autophagy. Autophagy (Greek for “the eating of oneself”) is an evolutionarily conserved lysosomal pathway that allows eukaryotic cells (yeast to mammals) to survive under nutrient starvation conditions [24–26]. Macroautophagy (herein referred to as autophagy) involves the bulk lysosomal turnover of long-lived proteins, protein aggregates, and organelles such as damaged mitochondria [27], damaged endoplasmic reticulum (ER) [28], and ribosomes [29]. The autophagic process occurs in several stages that begin with the formation of a crescent-shaped isolation membrane (phagophore) that sequesters organelles, matures into an autophagosome that surrounds the organelle, followed by the fusion of the autophagosome with a lysosome to form an autophagolysosome [30, 31]. Hydrolytic enzymes within the acid pH of the interior of the autophagolysosome, then act to degrade macromolecules, thereby providing nutrients for the survival of the eukaryotic cell [26]. An analysis of this morphologic process at the molecular level reveals a complex series of biochemical events involving the products of numerous autophagy-related genes [24, 26, 32–35]. The autophagy pathway is becoming increasingly recognized as an important mechanism of tumor cell survival and drug resistance in cancer chemotherapy [36–38]. A recent study indicated that autophagy is activated in colorectal cancer cells and contributes to tolerance to nutrient deprivation [39]. We now show, for the first time, that deoxycholate (DOC) activates the autophagic pathway in noncancer colon epithelial cells (NCM-460), and that this activation contributes to cell survival. We also show that the constitutive activation of autophagy contributes to the survival of apoptosis-resistant colon cancer cells (HCT-116RC) that were developed in our laboratory by repeated exposure to increasing concentrations of DOC [40].

The present findings, coupled with findings from our in vivo animal model of deoxycholate-induced colonic inflammation [17] and from our in vitro apoptosis-resistant colon cancer cell lines [40], implicate autophagy in colon carcinogenesis and suggest an additional mechanism by which hydrophobic bile acids contribute to colon carcinogenesis. These studies may aid in the identification of potential biomarkers of the autophagy pathway in the nonneoplastic colonic mucosa of patients at risk for colon cancer. In addition, combining inhibitors of autophagy with chemotherapeutic agents commonly used to treat colon cancer may lead to an improved clinical outcome.

## 2. Materials and Methods

### 2.1. Chemicals

Sodium deoxycholate (DOC), 3-methyladenine (3-MA), rapamycin (Rapa) (derived from Streptomyces hygroscopicus), CuDIPS [copper(II) 3,5-diisopropylsalicylate hydrate), and catalase (10 000–40 000 U/mg protein) were obtained from Sigma-Aldrich (St. Louis, Mo, USA). Bafilomycin A_1_, MnTBAP [Mn (III) tetrakis (4-benzoic acid) porphyrin chloride], HBED [N,N′-Di-(2-hydroxybenzyl)ethylenediamine-N,N′-diacetic acid)], pepstatin A, and E-64d were from BIOMOL Research Laboratories (Plymouth Meeting, Pa, USA), hydroxychloroquine sulfate (HCS) was from Acros Organics (Morris Plains, NJ), and trypan blue was from Gibco BRL Life Technologies (Grand Island, NY, USA). The concentrations used to modulate autophagy and cell death in the cell culture experiments are provided below. In all instances, the specific concentrations that were reported in the literature were tested for their effect on the viability of NCM-460 and HCT-116RC cells used in the present study. In some instances dose-response curves that assess the concentration of the drug and its effect on cell viability were performed to ensure that the concentration of the chemical itself was not too toxic to be used in the proposed experiments. This was especially important for 3-MA, which is used at a wide range of millimolar concentrations in published reports. The three antioxidants (HBED, MnTBAP, CuDIPS) used to evaluate the DOC-induced increase in beclin-1 expression were used at the same concentration (100 *μ*M) so that direct comparisons can be made. The concentration chosen was well within the range of concentrations reported in the literature for use with cultured cells (see cited articles below), and this concentration did not induce any cytotoxicity in the sensitive NCM-460 cells. CuDIPS: 100 *μ*M [15]; Bafilomycin A_1_: 1 nM [41, 42]; Catalase: 1000 U/mg; E-64d: 10 *μ*g/mL [43]; 3-MA: 4 mM; HBED: 100 *μ*M [44, 45]; HCS: 10 *μ*M [46]; MnTBAP: 100 *μ*M [47]; Pepstatin A: 10 *μ*g/mL [43]; Rapa: 100 *μ*M.

### 2.2. Cell Lines and Tissue Culture Conditions

The NCM-460 colon cell line was obtained from INCELL Corporation, LLC (San Antonio, Tex, USA) and cultured in M3:10TM medium to insure that it maintains its characteristic features. This cell line was not obtained from a colon cancer and is considered to be a noncancerous colonic epithelial cell line. HCT-116RC is a stable apoptosis-resistant colon cancer cell line that was developed in our laboratory after persistent exposure to increasing concentrations of NaDOC [40]. This cell line was maintained in DMEM media supplemented with 10% heat-inactivated fetal calf serum (Omega Scientific, Tarzana, Calif, USA), 1% MEM nonessential amino acids, 100 *μ*g/mL streptomycin, 100 U/mL penicillin, and 3.44 mg/mL L-glutamine. Media components were from Gibco BRL Life Technologies (Grand Island, NY, USA). All treatments utilizing NCM-460 cells were performed with the cells in approximate mid-logarithmic phase to avoid inconsistencies in cellular responses based on growth phase, as previously described [48]. Starvation experiments were performed by incubation cells in Hank's Balanced Salt Solution (HBSS), as previously described for colon epithelial cells [49]. HBSS was obtained from Gibco/Invitrogen Corp., Carlsbad, Calif, USA.

### 2.3. Assessment of Vacuolar Acidification

NCM460 cells were plated on a fibronectin-coated Costar (Fisher Scientific, Pittsburg, Pa, USA) 96 well black plate at 2 × 10^5^ cells/mL. Cells were treated with 0.4 mM DOC for 30 minutes and 1 hour. After treatments, cells were spun in an Allegra 6R Centrifuge (Beckman Coulter, Fullerton, Calif, USA) at 1000 rpm for 5 minutes and the fluorescent dyes added as described below. Triplicate wells were plated for each experimental protocol, and the intensity values were normalized for each well using a nucleic acid stain. Mean fold changes in intensity values (DOC versus control untreated cells) were statistically compared using Student's *t*-test.

#### 2.3.1. Monodansylcadaverine (MDC)

MDC (Fluka-Sigma-Aldrich, St. Louis, Mo, USA) was used at a final concentration of 200 *μ*M, and cells were incubated for 30 minutes at 37°C. MDC was removed and Sytox Green (Molecular Probes/Invitrogen, Carlsbad, Calif, USA) was added at a final concentration of 2.5 *μ*M; cells were incubated for 20 minutes at room temperature. Fluorescence was assessed immediately using an Optima FLUOstar plate Reader (BMG Labtech, Durham, NC, USA). MDC (excitation 335 nm; emission 508 nm) and Sytox Green (excitation 504 nm; emission 523 nm) fluorescence were assessed using appropriate filters. All fluorescence values were normalized to the amount of cells in individual wells by obtaining the ratio of MDC fluorescence (assessment of acid vesicles) to Sytox Green fluorescence (nucleic acid stain).

#### 2.3.2. LysoTracker Red

LysoTracker Red (Molecular Probes/Invitrogen, Carlsbad, Calif, USA) was added to the cells at a final concentration of 100 nM and incubated for 30 minutes at 37°C. Lysotracker Red was removed and Hoechst # 33342 dye (Molecular Probes/Invitrogen, Carlsbad, Calif, USA), made up in tissue culture media at a final concentration of 10 *μ*g/mL, was added to the cells and incubated for 10 minutes at 37°C. Cells were spun at 1000 rpm for 10 minutes, the supernatant removed, and the pelleted cells were fixed with 4% formaldehyde in PBS for 20 minutes at room temperature. Fluorescence at 30 minutes and 1 hour was assessed immediately using an Optima FLUOstar plate Reader (BMG Labtech, Durham, NC, USA). LysoTracker Red (excitation 577 nm; emission 590 nm) and Hoechst (excitation 350 nm; emission 461 nm) fluorescence were assessed using appropriate filters. All fluorescence values were normalized to the amount of cells in individual wells by obtaining the ratio of LysoTracker Red fluorescence (assessment of acid vesicles) to Hoechst fluorescence (nucleic acid stain).

### 2.4. Western Blot Analysis

Cells were grown in 20 × 100 mm Falcon polystyrene tissue culture dishes (Fisher Scientific, Pittsburgh, Pa, USA). Cultures treated with DOC or incubated in control media were disrupted in lysis buffer (50 mM Tris pH 8, 5 mM EDTA, 150 mM NaCl, 0.5% NP-40) supplemented with 1 mM phenylmethylsulfonyl fluoride (PMSF), leupeptin (1 *μ*g/mL), and aprotinin (0.01 U/mL). Cell lysates were prepared at a concentration of 2 *μ*g/*μ*L of protein, and 10 *μ*g of protein were added to each well of a 15% Criterion Tris-HCl gel (Biorad, Hercules, Calif, USA) for size fractionation by electrophoresis. The proteins were blotted onto Immobilon-P PVDF transfer membrane (Millipore, Bedford, Mass, USA). The membranes were incubated with a mouse antibeclin monoclonal antibody (BD Transduction Laboratories, San Diego, Calif, USA) at a dilution of 1 : 1000 or rabbit anti-LC3B polyclonal antibody (Cell Signaling Technology, Inc., Boston, Mass, USA) at a dilution of 1 : 500. The membranes were then incubated with goat antimouse or goat antirabbit secondary antibodies conjugated to horseradish peroxidase (Pierce, Rockford, Ill, USA). Antibody complexes were detected using the SuperSignal West Pico chemiluminescence detection system (Pierce, Rockford, Ill, USA). Finally, the membranes were stained for 20 minutes with Brilliant Blue G dye (Sigma-Aldrich, St. Louis, Mo, USA) to confirm equal protein loading. We have chosen to use the staining of the membranes with Brilliant Blue G dye rather than a specific protein as a loading control, since the former looks at numerous bands, whereas the latter can be misleading. This is based on work published from our laboratory using GAPDH and G3PD [50], and those of others who screened 22 housekeeping genes [51] and found a large number to be modulated by various experimental conditions.

All western blot experiments were repeated at least three times; in the repeats, separate cultures were treated, and cell lysates were separately prepared. The band intensities after DOC treatments or starvation conditions (incubation in HBSS) were then statistically compared using automated densitometry (QuantiScan Imaging Analysis, Biology Software Net, UK) and the values normalized to the control values. Two replicates from the same lysate were also run to ensure technical reproducibility.

### 2.5. Immunohistochemistry Procedures

Cells were grown in 20 × 100 mm Falcon polystyrene tissue culture dishes. NCM-460 cells were treated with 0.2 mM DOC for 24 hours. Control (untreated) and DOC-treated cells were trypsinized, washed with PBS and fixed with 4% formaldehyde overnight. Cell pellets were paraffin-embedded, and 4 micron sections were prepared. The details of the immunohistochemical procedures have been previously described [52]. Briefly, after deparaffinization, rehydration, and incubation in 3% hydrogen peroxide in methanol, sections were blocked with 1.5% goat serum (Vector Laboratories, Burlingame) and immunostained using a polyclonal antibeclin-1 antibody from ProSci Inc. (Poway, Calif, USA) at a concentration of 1 *μ*g/mL. Sections were then incubated using a biotinylated antirabbit secondary antibody (Vector Laboratories), Vectastain Elite ABC (Avidin Biotin Complex) reagent (Vector Laboratories), and DAB (3-3′diaminobenzidine) activated by hydrogen peroxide. Sections were then counterstained with hematoxylin.

### 2.6. Cytotoxicity Assays

#### 2.6.1. Trypan Blue Exclusion Assay

NCM-460 and apoptosis-resistant HCT-116RC cells were plated at 2 × 10^5^ cells/mL in a 24 well Falcon plate. After treatments, the supernatants containing floaters were removed, adherent cells were trypsinized and added to the floaters. An equal volume of trypan blue solution was added to a 50 *μ*L aliquot of the supernatant containing adherent cells and floaters. A minimum of 100 cells were counted on a hemacytometer slide under the 10X objective of a brightfield microscope. The percentage of cells that were stained with trypan blue was determined for each treatment. Each experiment was performed at least twice.

#### 2.6.2. Quantitation of Apoptosis and Necrosis

Cells were spun onto Glass slides using the Cytospin 3 (Shandon, Pittsburg, Pa, USA) and then were fixed in 100% methanol for 3 minutes. To stain the slides, 10% Giemsa stain (Sigma) were added for 4 hours. Cells were examined under a 100 X oil immersion lens and evaluated for apoptosis and necrosis, using criteria previously reported for brightfield microscopy [21]. All cell death experiments were repeated at least twice with similar results. To evaluate statistical significance, 100 cells were scored from 5 different areas of the slide, and a mean ± S.D. was obtained for each experimental group. The difference between groups was considered significant at the 95% probability level using Student's *t*-test.

### 2.7. Transmission Electron Microscopy (TEM)

Cells were grown in 10 × 35 mm Falcon polystyrene tissue culture dishes (Fisher Scientific, Pittsburgh, Pa, USA). All cells were pretreated with protease inhibitors (10 *μ*g/mL E-64d, 10 *μ*g/mL pepstatin A) to enhance the identification of cellular organelles and debris in autophagic vacuoles. Cells were then incubated with 0.4 mM DOC for 1–3 hours. Control cells were incubated in the absence of DOC for the same period of time. A total of at least 3 × 10^6^ cells from the two kinds of treatment groups were rinsed in PBS and fixed in situ with 1% glutaraldehyde made up in 0.1 M cacodylate buffer (pH 7.2) for 30 minutes. Cells were then scraped from the surface of the tissue culture plates using a rubber policeman, pelleted, and then resuspended in 3% glutaraldehyde made up in 0.1 M cacodylate buffer (pH 7.2) for 2 hours at 4°C. Cells were washed in PBS, postfixed in 2% osmium tetroxide, dehydrated in a graded series of ethanols, and embedded in Spurr's epoxy resin. Ultrathin sections were stained with uranyl acetate and lead citrate and photographed using a Philips CM12S transmission electron microscope operating at 80 keV.

## 3. Results

### 3.1. DOC Activates the Autophagic Pathway in Noncancer Colonic Epithelial Cells (NCM-460)

**Figure 1:**
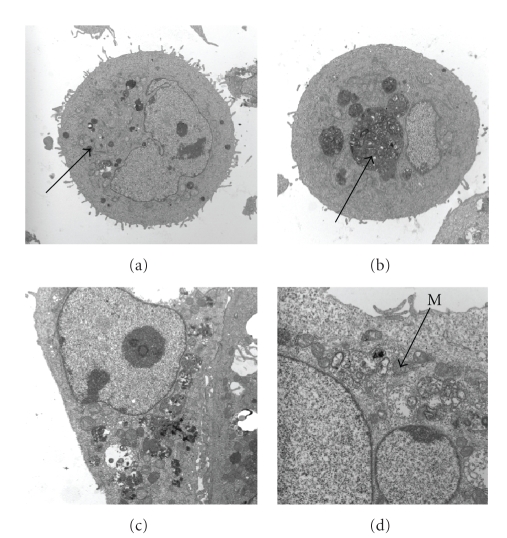
Transmission electron micrographs of control NCM-460 colonic epithelial cells (a) and cells treated with 0.4 mM DOC for 1 (b), 2 (c), and 3 (d) hours. Note the increase in number and size of autophagolysosomes after DOC treatment. (a) Arrow indicates the presence of small, electron-dense lysosomes; X4,400); (b) arrow indicates a large autophagolysosome with adjacent smaller autophagolysosomes in the process of fusing with the larger autophagolysosome (X7,100); (c) a large number of autophagic vacuoles containing cellular debris in various stages of degradation are present (X7,100); (d) arrow indicates the presence of a mitochondrion (M) within an autophagic vacuole (X15,000). (All cells were pretreated with protease inhibitors to retard the degradation process within lysosomes (see Section 2); this allowed the identification of cellular organelles that were difficult to observe in the absence of the protease inhibitors.) (Uranyl acetate, lead citrate counterstains.)

**Figure 2:**
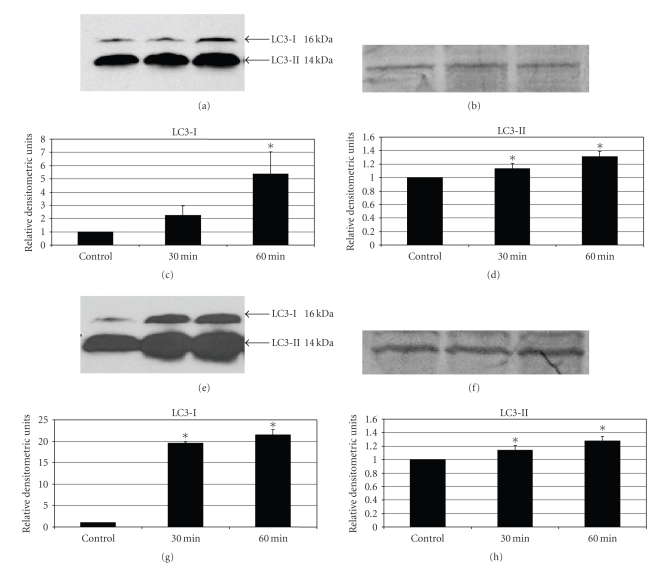
Western blot analysis of LC3-I and LC3-II protein expressions in NCM-460 cells in the presence of protease inhibitors (see Section 2) under normal conditions, after DOC treatment (a), (c), (d), and under starvation conditions (e), (g), (h). Bar graphs compare the protein expression of LC3-I and LC3-II over time using computerized densitometric analysis (c), (d), (g), (h). (a), (c), (d) represent LC3-I and LC3-II protein expressions after incubation with 0.4 mM DOC for 30 and 60 minutes compared to control cells that were not incubated with DOC. (e), (g), (h) represent LC3-I and LC3-II protein expressions at the same time points under starvation conditions using HBSS. The membranes were stained with Brilliant Blue G dye to confirm equal protein loading (Figures 2(b), 2(f)). (∗ indicates statistically significant (*P* < .05) differences in mean Relative Densitometric Units (RDU) between treatment and untreated control groups.)

Since NCM-460 cells are very sensitive to DOC-induced cytotoxicity, only 1–3 hour experiments were performed using 0.4 mM DOC; longer treatment times with this concentration of DOC resulted in significant apoptosis and necrosis. Treatment of NCM-460 cells with 0.4 mM DOC for 1–3 hours resulted in the appearance of autophagic vacuoles, assessed using TEM (Figure 1). These ultrastructural findings are considered one of the gold standards for identifying the activation of autophagy [53]. Another major finding was the increase in expression of microtubule-associated proteinlight chain 3 (LC3), the mammalian homologue of the yeast Atg8 autophagic protein [54]. The appearance of cytosolic LC3-I by posttranslational modification of Pro-LC3 by hATG4B [55] and the dynamic increase in the formation of LC3-II (a LC3-phospholipid conjugate) and localization to autophagosomal membranes [55] over time in the presence of protease inhibitors (E-64d and pepstatin A) are considered another excellent indication for the activation of the autophagic pathway [44]. The use of protease inhibitors prevents the degradation of LC3-II which is membrane-bound and subject to proteolytic degradation in mature autophagolysosomes. We preincubated NCM-460 cells in media containing 10 *μ*g/mL E-64d and 10 *μ*g/mL pepstatin A for 24 hours and then exposed cells to 0.4 mM DOC for 0-1 hour, a time period that we determined to have abundant autophagic vacuoles by TEM and prior to the appearance of apoptotic or necrotic cells. Figure 2 shows Western blots and densitometric analysis of DOC-treated NCM-460 cells (Figures 2(a)–2(d)) and starved cells (incubation in HBSS) as a positive control (Figures 2(e)–2(h)), indicating the dynamics associated with the appearance of LC3-I and LC-3-II. It can be seen that both LC3-I and LC3-II were increased by DOC and starvation conditions at the 1 hour time point compared to control untreated cells, indicating the activation of the early cytosolic form of LC3 (LC3-I) and the late membrane-bound form of LC3 (LC3-II). In different experiments the basal level of LC3-I was more variable than that of LC3-II. The reason for this is not clear, but may relate to different number of cell passages. In all cases, the basal levels of both LC3-I and LC3-II were increased by both DOC and starvation conditions; however, the actual fold increase cannot be directly compared because of this inherent variability.

As shown in Figures 2(e)–2(h), the increase of LC3-I and LC3-II over time in the presence of the protease inhibitors was observed under starvation conditions as was observed after incubation in 0.4 mM DOC (Figures 2(a)–2(d)). This increase in LC3-I and LC3-II levels after DOC treatment was similar to the findings of Ellington et al. [56] who studied soybean B-group triterpenoid saponin-induced autophagy in a colonic adenocarcinoma cell line (HCT-15). In the present study and that of Ellington et al. [56], this increase in expression of LC3-I and LC3-II was accompanied by the presence of autophagic vacuoles assessed by TEM, the classic gold standard for the activation of the autophagic pathway.

**Figure 3:**
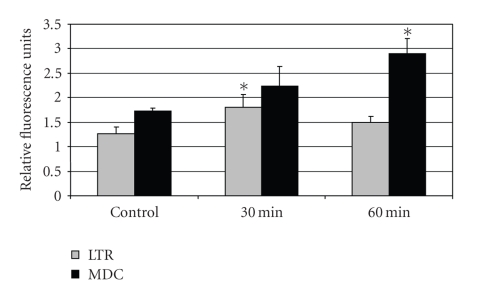
Effect of 0.4 mM DOC on the acidification of vesicles using MDC and Lysotracker Red (LTR) fluorescence assessed with a microplate reader. Note the statistically significant increase in vesicular acidification within 30 (LTR)–60 (MDC) minutes after DOC treatment. (∗ indicates statistically significant (*P* < .05) differences in mean Relative Fluorescence Units (RFU) between treatment groups and untreated control groups.)

An early step in the autophagic process is the acidification of cytoplasmic vesicles, which provides the acidic milieu necessary for the optimal activity of digestive enzymes contained within lysosomes. We were able to demonstrate the acidification of vesicles within 30 to 60 minutes after DOC treatment by assessing either the increase in fluorescence of MDC or Lysotracker Red (Figure 3), two dyes that target acid vesicles [57, 58]. The TEM studies coupled with the LC3 results and the vesicular acidification assays strongly indicate that hydrophobic bile acids can activate autophagy as an early stress-response pathway.

**Figure 4:**
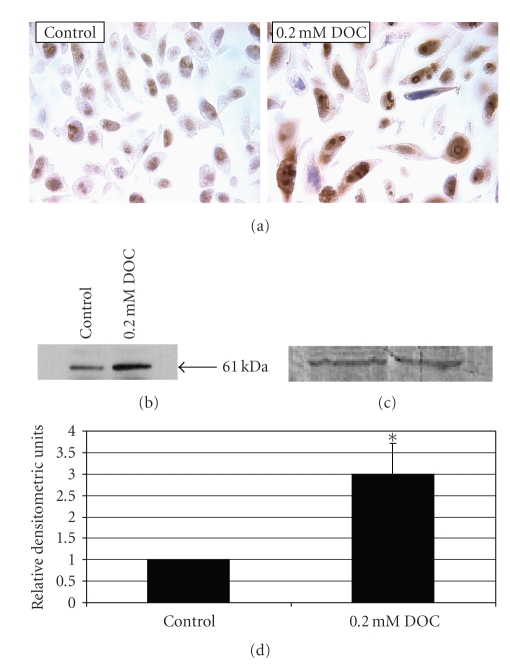
Immunohistochemical (a) and Western blot analysis (b), (c) of beclin-1 protein expression in NCM-460 cells in the absence and presence of 0.2 mM DOC for 24 hours. (a) There is a dramatic increase in the staining of beclin-1 in both the nucleus and cytoplasm of DOC-treated cells (right panel) compared with control untreated cells (left panel). Beclin-1 staining also occurs in association with the nucleolus where a ring of brown staining is observed in several cellular profiles in DOC-treated cells. (100X oil objective; brown color of DAB indicates positive beclin-1 staining; blue color indicates hematoxylin stain). (b) Western blot indicating the increase in the beclin-1 61 kDa band after DOC treatment. (c) The membranes were stained with Brilliant Blue G dye to confirm equal protein loading for western blot analysis. (d) Computerized densitometric evaluation of bands shown in (b). (∗ indicates statistically significant (*P* < .05) differences in mean Relative Densitometric Units (RDU) between treatment and untreated control groups.)

DOC also induced an increase in beclin-1, an essential autophagy protein [59]. NCM-460 cells were exposed to 0.2 mM DOC for 24 hours, and beclin-1 expression was assessed using immunohistochemical (Figure 4(a)) and Western blot (Figures 4(b)–4(d)) analysis. This concentration of DOC did not induce appreciable apoptosis during a 24-hour period and was, therefore, chosen for this experiment. Treatment with 0.2 mM DOC induced a dramatic increase in the protein levels of beclin-1 using both techniques.

### 3.2. DOC-Induced Increase in Beclin-1 Expression Is Mediated through an Oxidative Stress Mechanism

**Figure 5:**
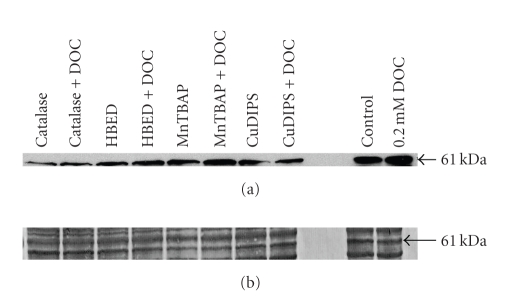
Effect of antioxidants and an antioxidant enzyme on protein expression of beclin-1. Cells were pretreated for 2 hours with either catalase, HBED, MnTBAP, or CuDIPS, followed by a 24-hour incubation with 0.2 mM DOC. (a) All pretreatments decreased both the constitutive beclin-1 protein levels and the DOC-induced increase in beclin-1 protein levels, with catalase being the most effective. (b) The Brilliant Blue loading control with the position of the beclin-1 band indicated by the arrow.

Since DOC induces a significant amount of oxidative/nitrosative stress [6, 11–15], we determined if the DOC-induced increase in beclin-1 expression was mediated, in part, through an oxidative mechanism. We pretreated NCM-460 cells for 2 hours with 4 different agents that reduce oxygen-free radicals through different mechanisms, followed by a 24-hour incubation with 0.2 mM DOC. The 4 agents used were catalase, HBED, MnTBAP, and CuDIPS. Catalase catalytically breaks down hydrogen peroxide to water and oxygen [60]; HBED is an iron chelator and inhibits ferric ion catalyzed formation of hydroxyl radicals [45]; MnTBAP is a cell permeable superoxide dismutase mimetic (SOD) and peroxynitrite scavenger [61, 62]; CuDIPS is a cell permeable SOD mimetic [63]. All 4 agents had a marked effect on preventing the DOC-induced increase in beclin-1 expression, although catalase was the most effective (Figure 5). In addition, it was determined that the constitutive levels of beclin-1 are also highly dependent on endogenous oxidative stress levels in the cell. As shown in Figure 5, all 4 antioxidants decreased the constitutive levels of beclin-1, with catalase being the most effective.

### 3.3. Autophagy Protects NCM-460 Cells from DOC-Induced Cell Death

**Figure 6:**
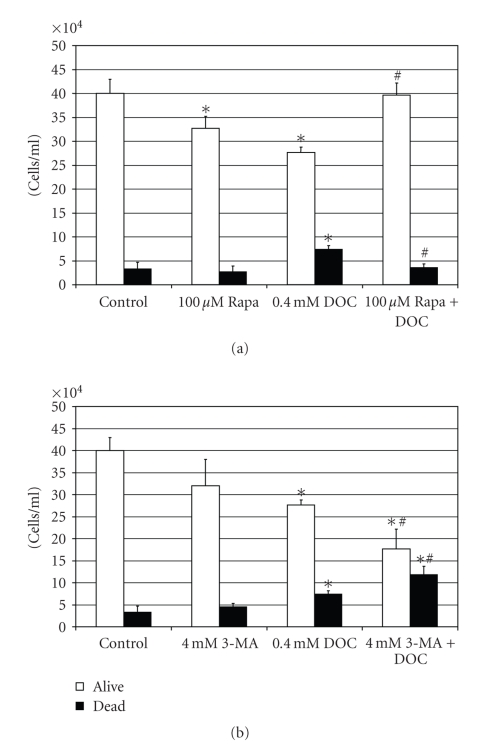
Bar graphs demonstrating the effects of 0.4 mM DOC and 100 *μ*M rapamycin pretreatment (a) or 0.4 mM DOC and 4 mM 3-MA (3-methyladenine) pretreatment (b) on cell number and viability (trypan blue exclusion) of NCM-460 cells. (Significant differences (*P* < .05) between treatment groups and control (untreated) cells are indicated by an asterisk (∗). Significant differences between DOC treatment, alone, and DOC treatment after rapamycin or 3-MA pretreatment are indicated by a pound sign (#).)

To determine whether the activation of autophagy contributes to DOC-induced cell death or is a prosurvival stress-response pathway, NCM-460 cells were pretreated with rapamycin, an agent that activates autophagy [64], and 3-methyladenine (3-MA), an agent that inhibits the autophagic process [65]. NCM-460 cells were pretreated with 100 *μ*M rapamycin (Figure 6(a)) or 4 mM 3-MA (Figure 6(b)) for 24 hours and then incubated with 0.4 mM DOC for 4 hours. Total cell number and the trypan blue exclusion assay were used as measures of cell growth and viability.

DOC treatment, alone, resulted in a significant (*P* < .05) decrease in cell counts compared to untreated control cells. Rapamycin pretreatment significantly (*P* < .05) decreased trypan blue uptake and prevented the cell loss caused by DOC treatment (Figure 6(a)). The significant decrease in cell counts in the absence of significant trypan blue uptake by 100 *μ*M rapamycin, alone (Figure 6(a)), is most probably a reflection of a decrease in cell proliferation caused by the activation of autophagy. Opposite to the effects of rapamycin, pretreatment with 3-MA significantly (*P* < .05) increased trypan blue uptake and increased the cell loss caused by DOC (Figure 6(b)).

### 3.4. Autophagy Protects HCT-116RC Apoptosis-Resistant Colon Cancer Cells from DOC-Induced Cell Death

We have previously reported that persistent exposure of HCT-116 apoptosis-competent colon cancer cells to increasing concentrations of DOC resulted in the development of stable apoptosis-resistant cell populations in which several stress-response pathways were upregulated [40, 66]. It was determined that the autophagic activity was constitutively upregulated in each of the apoptosis-resistant cell lines (HCT-116RB, HCT-116RC, HCT-116RD cells). Increased autophagy was indicated by the presence of numerous late-stage autophagolysosomes in the cytoplasm of the resistant cells, identified in some cases by the presence of numerous whorls of digested material [40].

**Figure 7:**
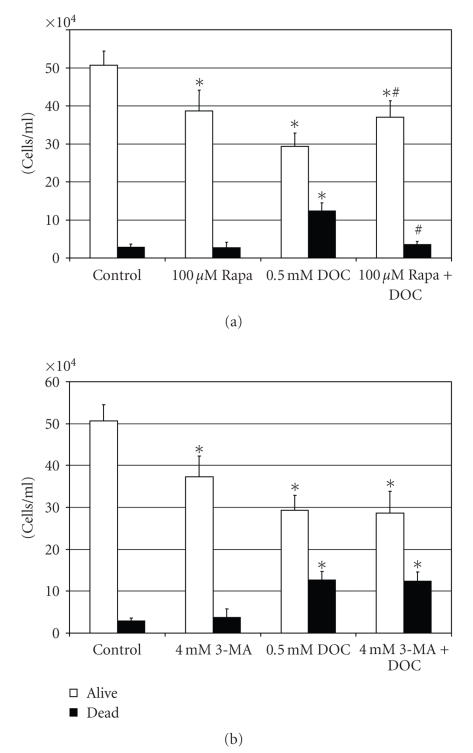
Bar graphs demonstrating the effects of 0.5 mM DOC and 100 *μ*M rapamycin pretreatment (a) or 0.5 *μ*M DOC and 4 mM 3-MA (3-methyladenine) pretreatment (b) on cell number and viability (trypan blue exclusion) of HCT 116RC apoptosis-resistant cells. (Significant differences (*P* < .05) between treatment groups and control (untreated) cells are indicated by an asterisk (∗). Significant differences between DOC treatment, alone, and DOC treatment after rapamycin or 3-MA pretreatment are indicated by a pound sign (#).)

To evaluate whether the constitutive upregulation of the autophagic pathway has a survival function in these apoptosis-resistant cells or is merely an epiphenomenon, we exposed HCT-116RC cells to various agents that modulate the autophagic process. Since the HCT-116RC cells are resistant to cell death, all experiments requiring bile acid treatment were performed using 0.5 mM DOC, and cells were treated in late log phase of growth. These conditions were necessary to elicit a cellular response to autophagy inhibitors/inducers, as described below. HCT-116RC cells were pretreated with 100 *μ*M rapamycin or 4 mM 3-MA for 24 hours and then incubated with 0.5 mM DOC for an additional 24 hours. Total cell number and the trypan blue exclusion assay were used as measures of cell growth and viability. Similar to the results with the noncancer cell line, NCM-460, rapamycin pretreatment of the apoptosis-resistant cancer cell line, HCT-116RC, followed by 24 hours of treatment with 0.5 mM DOC, resulted in a significant (*P* < .05) increase in cell number and a significant (*P* < .05) decrease in trypan blue uptake (i.e., increase in viable cells) compared to DOC treatment, alone (Figure 7(a)). The significant decrease in cell counts in the absence of significant trypan blue uptake by 100 *μ*M rapamycin, alone (Figure 7(a)), is most probably a reflection of a decrease in cell proliferation caused by the activation of autophagy. On the other hand, pretreatment of HCT-116RC cells with 4 mM 3-MA had no effect on increasing cell death induced by 0.5 mM DOC (Figure 7(b)). The significant decrease in cell counts in the absence of significant trypan blue uptake by 4 mM 3-MA, alone, is most probably a reflection of a decrease in cell proliferation (Figure 7(b)).

Since we have previously shown that autophagy is constitutively expressed in these cells [40] and rapamycin had a significant effect on cell survival, the negative results obtained with the combination of 3-MA and DOC cannot be taken as conclusive evidence of lack of involvement of autophagy [58]. Since 3-MA inhibits autophagy at an early stage by preventing the formation of autophagosomes, it has been reported that in order to adequately assess the modulation of the autophagy process, inhibitors that act at a different stages of the autophagy process should also be tested [58]. Therefore, HCT-116RC cells were exposed to 2 different inhibitors of the autophagic process, bafilomycin A_1_ and hydroxychloroquine, which act at the level of acid vesicles/lysosomes [67]. Bafilomycin A_1_ appears to block the fusion of autophagosomes and lysosomes [68], and hydroxychloroquine (an amine) diffuses into acid vesicles/lysosomes and raises the intraorganellar pH. Bafilomycin A_1_ also raises the pH of acid vesicles/lysosomes by inhibiting the proton-translocating ATPase (H^+^-ATPase) [69]. HCT-116RC cells were pretreated with 1 nM bafilomycin A_1_ for 24 hours and then incubated with 0.5 mM DOC for an additional 24 hours. Bafilomycin A_1_ pretreatment followed by DOC treatment increased the percentage of apoptotic cells 4-fold over the level of apoptosis induced when DOC was used alone (Table 1). There was no increase in the percentage of DOC-induced necrotic cells by bafilomycin A_1_ pretreatment. HCT-116RC cells were pretreated with 10 *μ*M hydroxychloroquine for 24 hours and then incubated with 0.5 mM DOC for an additional 24 hours. Hydroxychloroquine pretreatment followed by DOC treatment increased the percentage of apoptotic cells 4-fold over the level of apoptosis induced by DOC, alone (Table 1(b)). There was no increase in the percentage of DOC-induced necrotic cells by hydroxychloroquine pretreatment.

In summary, the collective data indicate that autophagy has a survival value for both noncancerous and cancerous colon cells when exposed to hydrophobic bile acids in a nutrient-rich environment.

## 4. Discussion

**Figure 8:**
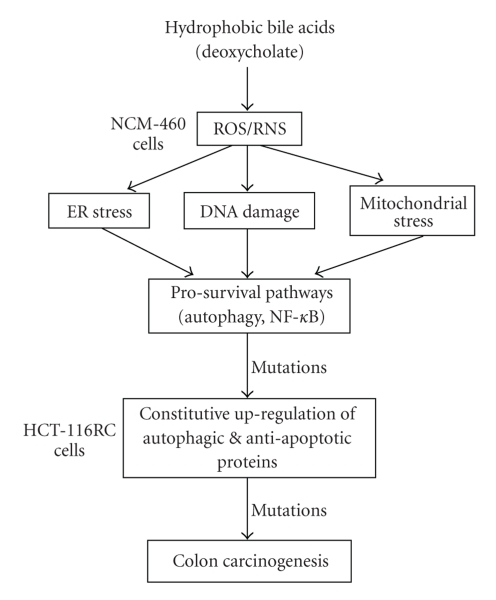
Diagram indicating the possible roles of autophagy in colon carcinogenesis. Hydrophobic bile acids are known to induce numerous stresses in colon epithelial cells that result in the activation of prosurvival stress-response pathways. NF-*κ*B activation by hydrophobic bile acids induces prosurvival pathways. The present study reports that deoxycholate (DOC), a hydrophobic bile acid that is important in colon carcinogenesis, activates autophagy. This activation of autophagy by DOC was also shown to have a prosurvival function. The constitutive upregulation of prosurvival pathways (resulting from chronic exposure to DOC and selection for apoptosis resistance) can enhance mutation rates, which may lead to the development of colon cancer. NCM-460 and HCT- 116RC cells were used as model cell lines to evaluate the bile acid-induced activation of the autophagic pathway and its consequences in the early and late stages of colon carcinogenesis, respectively.

High concentrations of hydrophobic bile acids, associated with a high-fat diet, induce proapoptotic and prosurvival stress-response pathways [13]. The ultimate fate of the cell depends upon the balance of proapoptotic and antiapoptotic proteins activated or synthesized in response to bile acid exposure, and the level of energy demands placed upon the stressed cell [9]. We hypothesized that persistent cellular stress induced by bile acids, such as ER stress, DNA damage, and mitochondrial stress, will lead to the clonal selection of apoptosis-resistant cells and the constitutive activation of cell survival pathways (Figure 8). We tested this hypothesis by generating apoptosis-resistant colon cells by repeated exposure of apoptosis-sensitive HCT-116 cells in vitro to increasing concentrations of the hydrophobic bile acid, DOC, and evaluating stress-induced cell death and apoptosis-related gene expression at the molecular and cellular levels [40, 66]. NF-*κ*B and many proteins that protect against oxidative stress were constitutively upregulated in these apoptosis-resistant cells. In addition, the development of apoptosis resistance was accompanied by the modulation of genes associated with the autophagy pathway. The autophagy-related genes that exhibit increased expression include six rab genes involved in vesicle transport, a Rab interacting lysosomal protein-like 2 protein (RILPL2), PI(3)K, 2 subunits of the lysosomal proton (H^+^)-translocating ATPase, cathepsin D, lysosomal-associated membrane protein 1 (Lamp-1), a multipass membrane transporter protein (MFSD8/CLN7), and prenylcysteine lyase, a lysosomal enzyme involved in the degradation of prenylated proteins. We also found that chronic feeding of wild-type B6.129 mice with DOC added to the diet results in an increase in APG4 [17], a cysteine protease that acts during the formation of autophagosomes [70] and whose activity is regulated by reactive oxygen species (ROS) [71]. The functional role of autophagy in colon carcinogenesis, however, was not determined from these in vitro microarray and in vivo animal studies.

In the present study, we first evaluated the ability of DOC to activate autophagy in NCM-460 cells, and then determined whether autophagy has a prosurvival function in this noncancerous colon epithelial cell line. We demonstrated that DOC activated autophagy using different methods of detection, and that this activation contributed to cell survival. We next determined that the constitutive upregulation of autophagy also has a prosurvival function in HCT-116RC apoptosis-resistant colon cancer epithelial cells. The prosurvival mechanism of DOC-induced autophagy is probably antiapoptotic. This is based on the experiments with bafilomycin A_1_/hydroxychloroquine, in which we showed that autophagy prevented cells from undergoing DOC-induced apoptosis, but not from DOC-induced necrosis.

The possible roles of autophagy in colon carcinogenesis based on published results and present findings are shown schematically in Figure 8. The cellular stresses induced by hydrophobic bile acids (e.g., ER stress, DNA damage, mitochondrial stress) are also inducers of the autophagic pathway [72–75], most probably mediated through the generation of ROS [76]. Evidence that DOC induces the autophagic pathway through an oxidative/nitrosative mechanism was provided in the present study using 3 different antioxidants in addition to catalase. These antioxidant conditions dramatically reduced the level of DOC-induced beclin-1 protein expression, a major protein involved in the mammalian autophagic pathway.

The rationale for choosing beclin-1 (homologue of the yeast autophagy gene apg6/vps30 [77]) to assess the effects of antioxidants on the DOC-induced increase in autophagy was based on (1) its dramatic increase in expression in noncancer cells by DOC, a known inducer of oxidative stress, compared to other autophagy-related proteins (data not shown), (2) its critical involvement in the initial step of autophagosome formation [78–80], (3) the documented importance of an increase in beclin-1 at the premetastasis stage of colon cancer development [81], (4), its role in tumorigenesis, in general [82, 83], (5) its function as an antiapoptotic protein [84–87], and (6) its potential as a possible biomarker to assess colon cancer risk. Although the oxidative mechanism by which DOC increases beclin-1 protein expression is most probably multifactorial, we suggest that an important signaling pathway may involve the generation of ceramide. This is based on the fact that (1) ceramide is an important sphingolipid molecule involved in the increase in beclin-1 in HT-29 colon epithelial cells [88] and other cell types [89], DOC is known to generate ceramide through several mechanisms [80–92], (2) ceramide treatment decreases catalase enzymatic activity and expression [93], a possible link to the present findings indicating that catalase reduces the DOC-induced increase in beclin-1 expression, and (3) ceramide can damage mitochondria [94–98], a known inducer of the autophagic process [99]. Other mechanisms that may be responsible for DOC-induced increase in beclin-1 expression may involve alterations of lipid trafficking [100], a process known to induce beclin-1 expression in other cell types [101]. Although we focussed on the role of oxidative stress in the DOC-induced modulation of beclin-1, other aspects of the autophagic process that are now known to be regulated by ROS/RNS [102–105] may also be modulated by DOC.

Since we have shown that autophagy is a survival pathway for apoptosis-resistant colon cancer cells, the constitutive activation of autophagy and the activation of autophagy induced by cancer chemotherapeutic agents [106] should, therefore, be taken into consideration when designing effective clinical treatment regimens for cancer. We plan to determine the effectiveness of modulators of autophagy in combination with cytotoxic drugs, such as 5-fluorouracil, oxaliplatin, and irenotecan [107, 108], in enhancing cell death in vitro in our apoptosis-resistant colon cancer cell lines. The precedence for combining inhibitors of autophagy with chemotherapeutic agents for the treatment of colon was recently established [106]. Li et al. [106] reported that 3-MA enhanced the effect of 5-fluorouracil in inducing apoptosis of colo26 and HT-29 colon cancer cells. It is also anticipated that a better understanding of the mechanisms of autophagy in colon cells, their modulation by dietary factors, and aberrant expression of autophagic proteins during colon carcinogenesis will contribute to the important field of hypothesis-driven biomarker development to assess colon cancer risk.

## Figures and Tables

**Table:** (a)

Experimental group	% apoptotic cells	% necrotic cells
	(mean ± SEM)	(mean ± SEM)
Control (untreated)	2.8 ± 1.0	3.0 ± 0.6
1 nM Bafilomycin A_1_	3.0 ± 0.4	4.6 ± 0.3
0.5 mM DOC	4.2 ± 0.9	27.0 ± 3.5*
Bafilomycin A_1_ + DOC	17.8 ± 3.2^∗*#*^	27.8 ± 5.5*

**Table:** (b)

Experimental group	% apoptotic cells	% necrotic cells
	(mean ± SEM)	(mean ± SEM)
Control	0.4 ± 0.3	2.4 ± 0.3*
10 *μ*M Hydroxychloroquine	3.8 ± 1.0*	6.4 ± 0.6*
0.5 mM DOC	5.4 ± 0.9*	22.4 ± 4.3*
Hydroxychloroquine + DOC	20.0 ± 1.7^∗*#*^	19.8 ± 2.6*

*Statistically significant (*P* < .05) differences in mean values when compared to untreated control cells; # statistically significant (*P* < .05) differences in mean values when compared to cells treated with DOC, alone, and this goes for Tables 1(a) and 1(b).
